# Supplementary material for: Identification of photoperiod-induced specific miRNAs in the adrenal glands of Sunite sheep (Ovis aries)
Source: Front Vet Sci. 2022 Jul 22;9:888207. doi: 10.3389/fvets.2022.888207 (PMC9354845; doi:10.3389/fvets.2022.888207)
Supplement: Supplementary file 1 [file Data_Sheet_1.ZIP › Supplementary file/miRDeep2 file.zip!/miRDeep2 file/Know_Structure/SPLP42A3/oar-mir-21.pdf]

miRBase precursor : oar-mir-21
Total read count : 158749
oar-miR-21 read count : 158742
remaining reads : 7

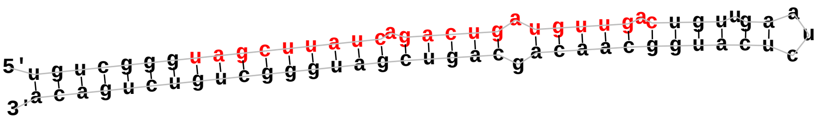

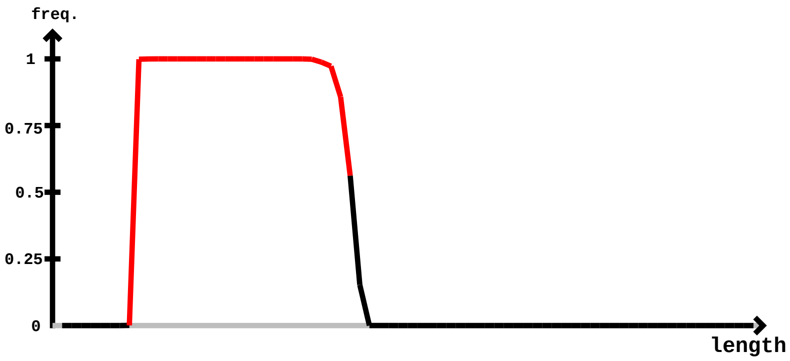


```
        oar-miR-21
5'- ugucggguagcuuaucagacugauguugacuguugaaucucauggcaacagcagucgaugggcugucugaca   -3'    exp
    ((((((((((((((((.(((((.(((((.((((.((...))))))))))).)))))))))))))))))))))    reads      mm         sample
    ......guagcuuaucagacugaugu..............................................    7          0          seq
    ......guagcuuaucagacugauguu.............................................    8          0          seq
    ......guagcuuaucagacugauguug............................................    32         0          seq
    ......guagcuuaucagacugauguuga...........................................    38         0          seq
    ......guagcuuaucagacugauguugac..........................................    14         0          seq
    ......guagcuuaucagacugauguugacu.........................................    5          0          seq
    .......uagcuuaucagacugau................................................    9          0          seq
    .......uagcuuaucagacugaug...............................................    192        0          seq
    .......uagcuuaucagacugaugu..............................................    1717       0          seq
    .......uagcuuaucagacugauguu.............................................    2398       0          seq
    .......uagcuuaucagacugauguug............................................    18221      0          seq
    .......uagcuuaucagacugauguuga...........................................    46862      0          seq
    .......uagcuuaucagacugauguugac..........................................    64816      0          seq
    .......uagcuuaucagacugauguugacu.........................................    24213      0          seq
    .......uagcuuaucagacugauguugacug........................................    3          0          seq
    .......uagcuuaucagacugauguugacugu.......................................    1          0          seq
    .......uagcuuaucagacugauguugacuguu......................................    9          0          seq
    .......uagcuuaucagacugauguugacuguug.....................................    3          0          seq
    .......uagcuuaucagacugauguugacuguugaau..................................    1          0          seq
    ........agcuuaucagacugaugu..............................................    1          0          seq
    ........agcuuaucagacugauguu.............................................    5          0          seq
    ........agcuuaucagacugauguug............................................    17         0          seq
    ........agcuuaucagacugauguuga...........................................    29         0          seq
    ........agcuuaucagacugauguugac..........................................    76         0          seq
    ........agcuuaucagacugauguugacu.........................................    31         0          seq
    .........gcuuaucagacugauguug............................................    4          0          seq
    .........gcuuaucagacugauguuga...........................................    13         0          seq
    .........gcuuaucagacugauguugac..........................................    12         0          seq
    .........gcuuaucagacugauguugacu.........................................    4          0          seq
    .........gcuuaucagacugauguugacuguu......................................    1          0          seq
    ...........uuaucagacugauguuga...........................................    1          0          seq
    .............................................caacagcagucgaugggcu........    1          0          seq
    .............................................caacagcagucgaugggcuguc.....    5          0          seq
```
